# Supplementary material for: Optimizing marine macrophyte capacity to locally ameliorate ocean acidification under variable light and flow regimes: Insights from an experimental approach
Source: PLoS One. 2023 Oct 11;18(10):e0288548. doi: 10.1371/journal.pone.0288548 (PMC10566731; doi:10.1371/journal.pone.0288548)
Supplement: S1 File — (PDF) [file pone.0288548.s001.pdf]

Supporting Information to "Optimizing marine macrophyte capacity to locally ameliorate ocean acidification under variable light and flow regimes: Insights from an experimental approach"

Aurora M. Ricart[1*], Brittney Honisch[1], Evangeline Fachon[2], Christopher W. Hunt[3], Joseph Salisbury[3], Suzanne N. Arnold[4], Nichole N. Price[1]

[1]Bigelow Laboratory for Ocean Sciences, 60 Bigelow Dr, East Boothbay, Maine 04544, USA

[2]Massachusetts Institute of Technology/Woods Hole Oceanographic Institution, 266 Woods Hole Road, Woods Hole, Massachusetts 02543, USA

[3]Ocean Process Analysis Laboratory, University of New Hampshire, Durham NH 03824, USA

[4]Island Institute, 386 Main St., Rockland, Maine 04841, USA

*** Correspondence:** Aurora M. Ricart. Email: aricart@bigelow.org

---

Figure S1. Photosynthetic active radiation (PAR) data from field collection sites. (**A**) PAR depth profile from the macroalgae collection site in the Damariscotta River Estuary Region, (**B**) time series (April to June 2018) of PAR data at 3 m depth and 5 km offshore from the seagrass collection in Broad Cove in Casco Bay. All PAR data was collected using MiniPAR sensors from Precision Measurement Engineering (PME).

A *B*

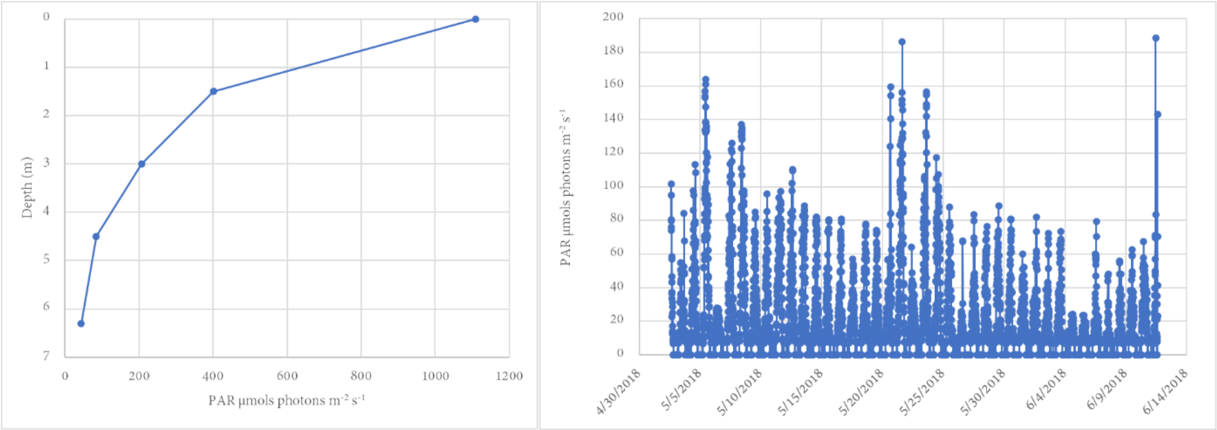

Figure S2. Conceptual diagram of the experimental set up based at the Bigelow facilities for Experiment 2 and used to examine the effects of residence time upon *S. latissima* capacity to ameliorate seawater acidity conditions under ambient and simulated future scenarios of climate change in a gradient of irradiance.

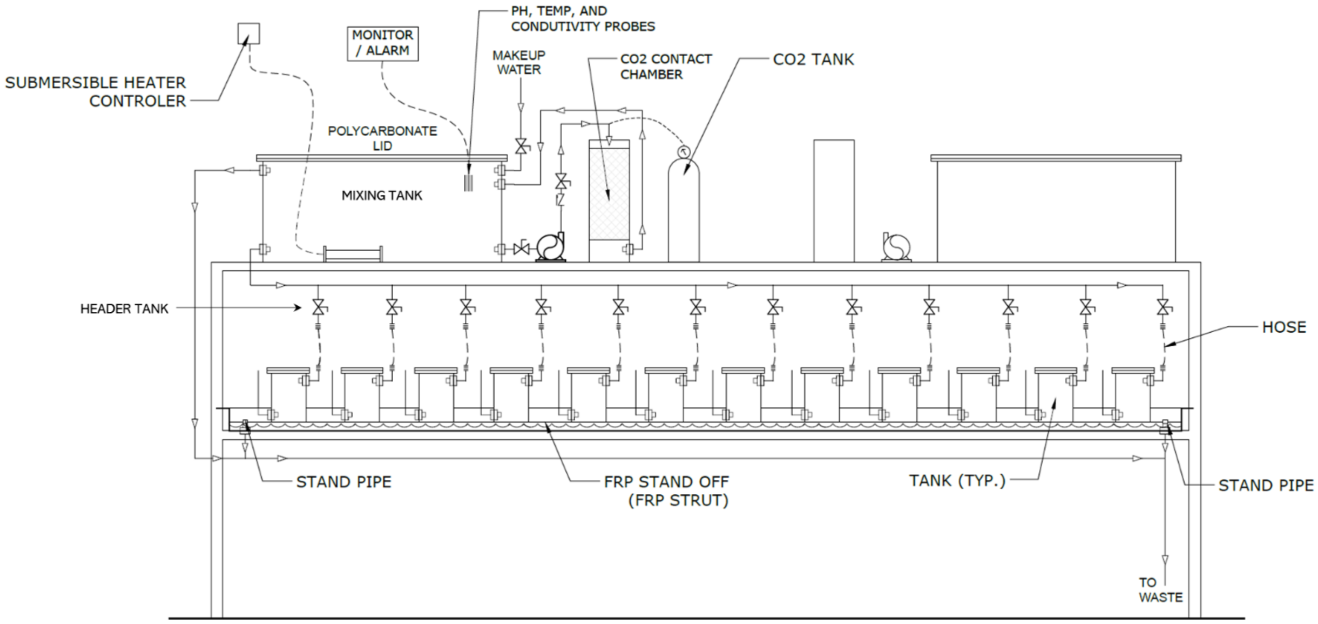

Figure S3. High frequency continuous measurements of dissolved oxygen (DO) in the flow & light experiment, Experiment 2. Measurements were made in one tank per treatment to decide the waiting time for measurements after light increases in each light step. Results showed that 60 min was enough to see stable changes in DO on each light step. Times of when light steps occurred are shown with vertical dashed lines. Symbols: simulated future environmental conditions and high residence time (blue- triangles); simulated future conditions and low residence time (red-circles); ambient conditions and high residence time (green-circles); ambient conditions and low residence time (purple-triangles).

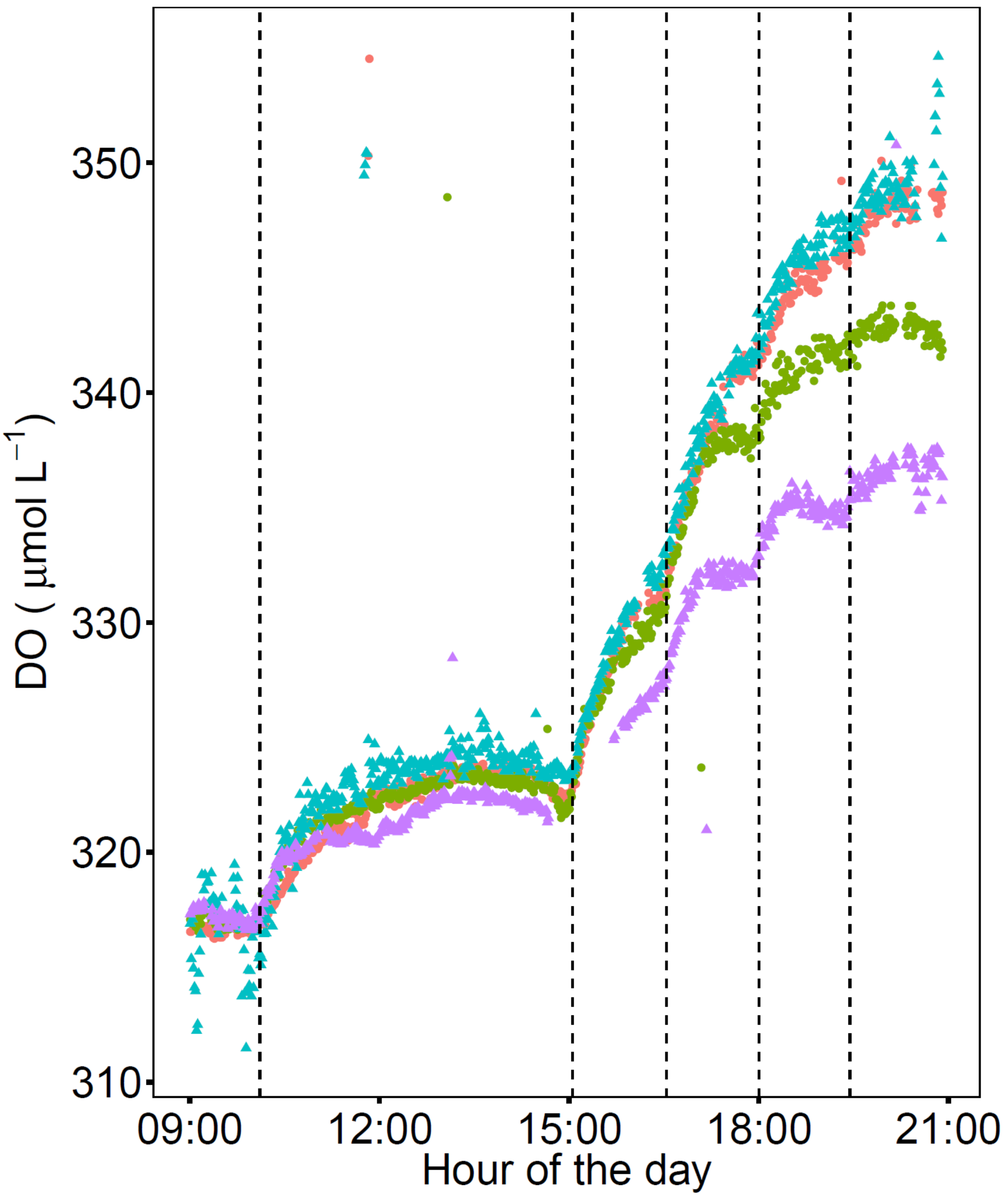

Figure S4. Speciation of inorganic carbon forms in the comparison among marine macrophyte species, Experiment 1. Plots show the level of change during incubations for (**A**) bicarbonate (HCO^{-}_{3}), (**B**) dissolved aqueous carbon dioxide (CO_2), (**C**) the carbonate ion (CO^{-2}_{3}), and (**D**) total dissolved inorganic carbon (DIC). Values reported are normalized per biomass of macrophyte, liter and hour. Plots include the levels in the control treatments with no macrophytes, that in this case are not normalized per macrophyte biomass. Colored lines show predicted average values and shade areas 95% CI in Controls (red-circles), *Sacharina latissima* (green-squares), *Ulva lactuca* (blue-crosses), *Zostera marina* (purple-cross within squares), and *Fucus vesiculosus* (yellow-triangles).

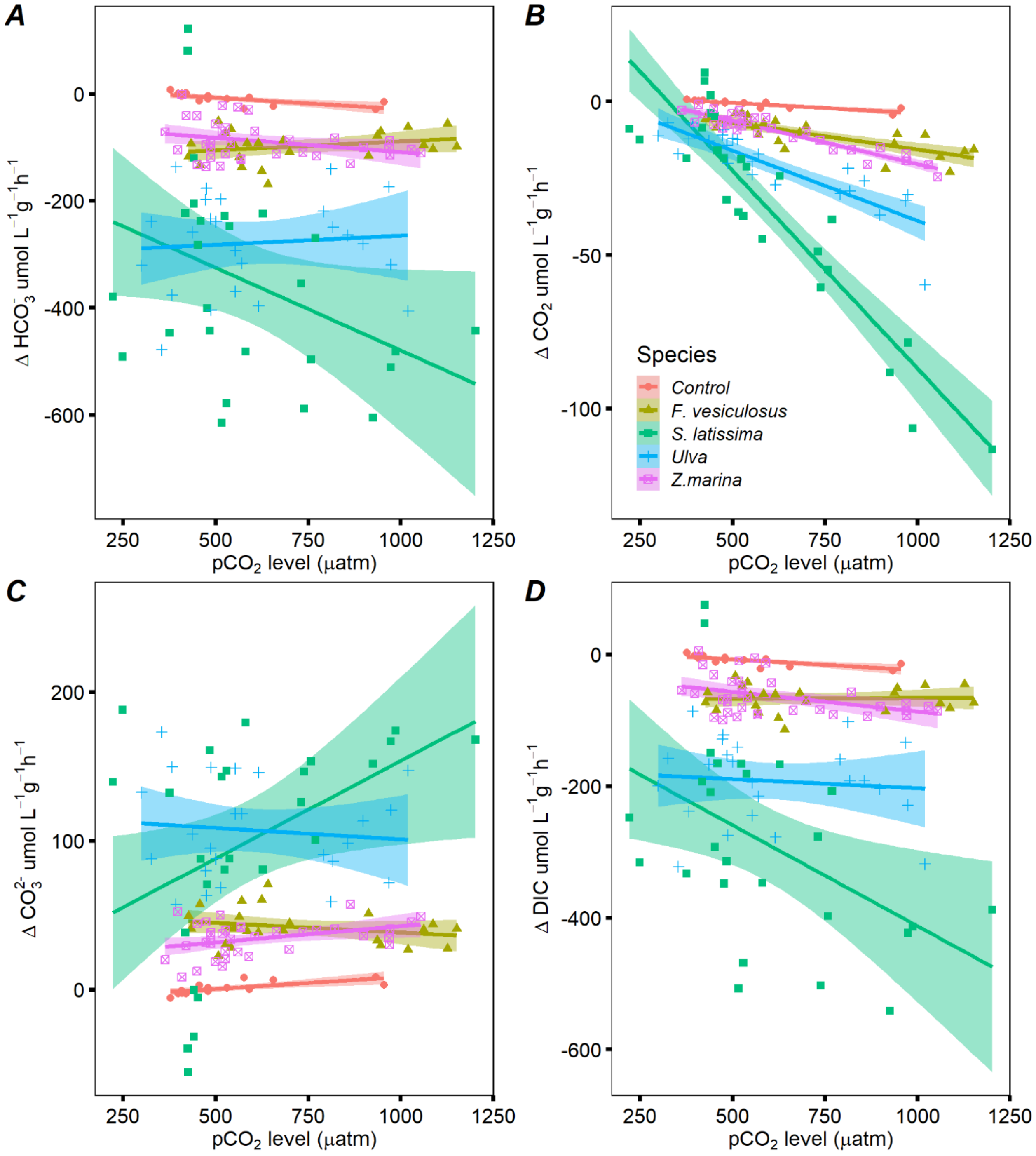

Figure S5. Speciation of DIC forms in the residence time and irradiance Experiment 2. Plots show level of change from the header tank for (**A, B**) bicarbonate (HCO^{-}_3), (**C, D**) dissolved aqueous carbon dioxide (CO_2), and (**E, F**) the carbonate ion (CO^{-2}_3). The black horizontal line represents the average value in the control treatments with no kelp. Colored lines show a smoothing with the loess method for values in low- (blue-triangles) and high- water flow (red-circles) and grey areas 95% CI.

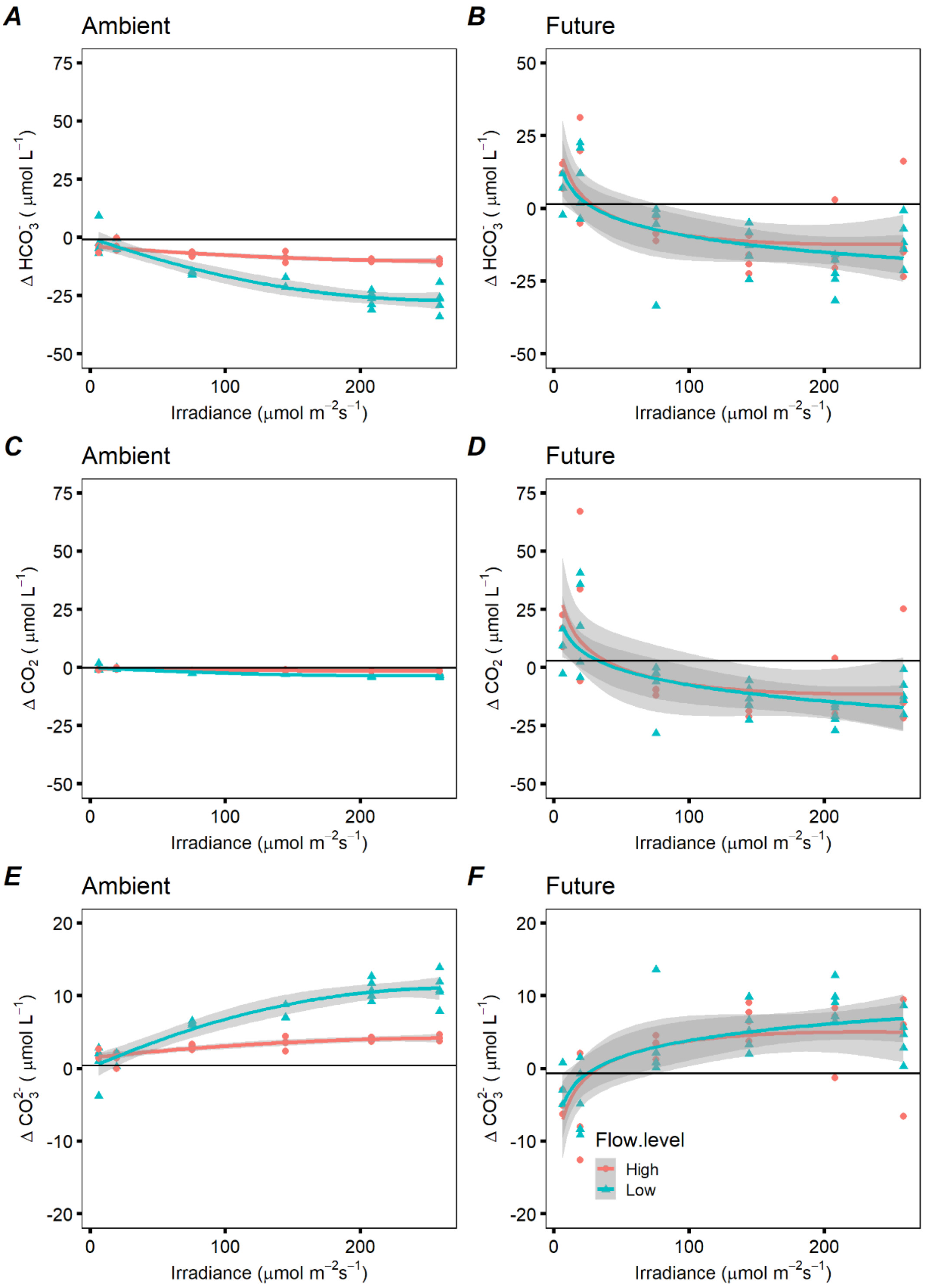

Figure S6. Average light curves per treatment derived from the fitted individual light curves of each tank. P represents the net rate of photosynthesis (ΔDO µmol L^{-1}) or capacity to ameliorate seawater acidity conditions (ΔDIC µmol L^{-1}, ΔΩ or ΔpH), E represents irradiance (µmol m^{-2} s^{-1}). For visualization, curves were derived up to E=1500 µmol m^{-2} s^{-1}, further models were done up to 250 µmol m^{-2} s^{-1}, as this was the maximum irradiance used during the Experiment 2.

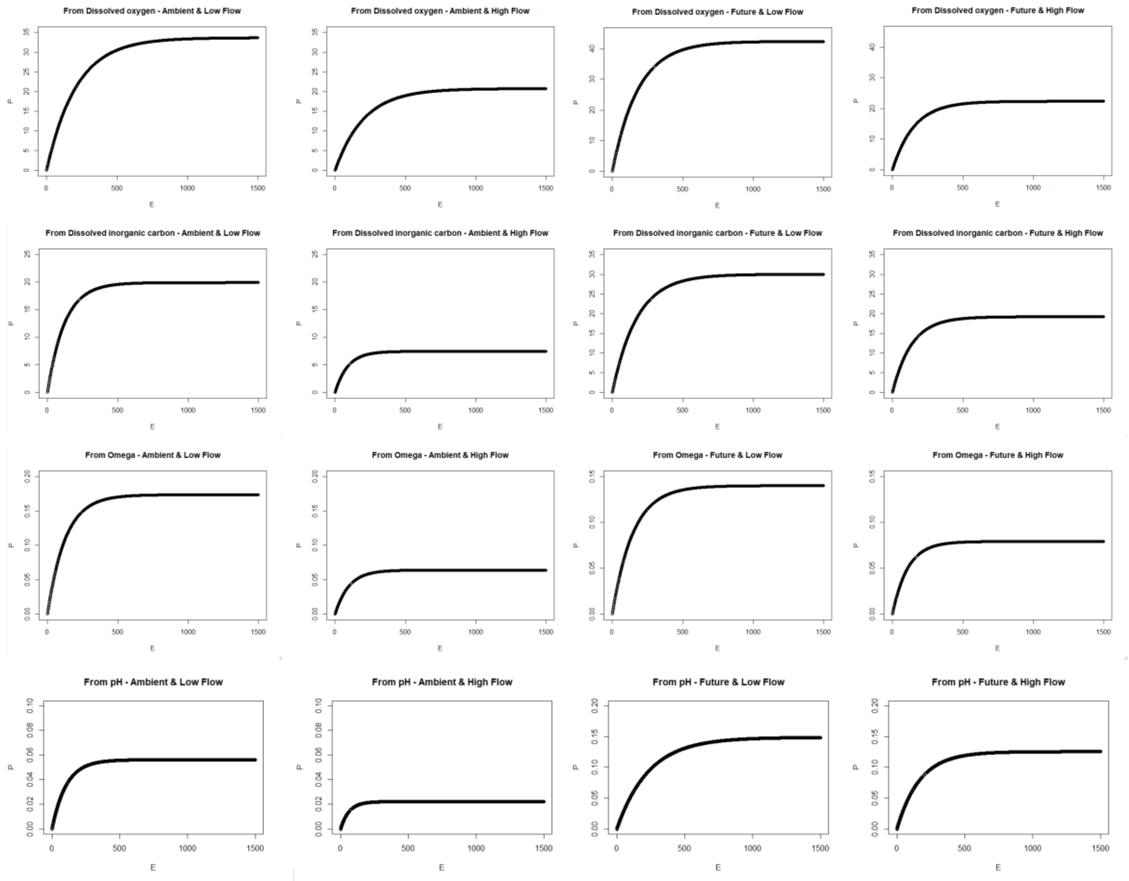

Table S1. Summary of the different parameters measured in the comparison among macrophytes species, Experiment 1. Values show mean ± SD. N, number of replicates; DO, dissolved oxygen; DIC, dissolved inorganic carbon; Ω saturation state of calcium mineral (aragonite).

							DO (µmol L^{-1})						DIC (µmol L^{-1})						pH						Ω_{ar}					
Species	pCO_2 Treatment (µatm)	N	Sal.	Temp.	Macrophytes biomass (g DW)		Before		After		ΔDO (µmol L^{-1}g^{-1}h^{-1})		Before		After		ΔDIC (µmol L^{-1}g^{-1}h^{-1})		Before		After		ΔpH (L^{-1}g^{-1}h^{-1})		Before		After		ΔΩ (L^{-1}g^{-1}h^{-1})	
Control	280	4	36.6	15.0	0	-	323.91	20.7	324.84	4.7	0.81	14.3	1896.30	19.6	1889.08	15.9	-4.14	3.3	7.97	0.1	7.96	0.0	0.00	0.0	1.91	0.2	1.87	0.1	-0.02	0.0
	400	2	37.0	14.2	0	-	329.84	2.4	320.00	1.8	-5.72	0.8	1879.66	5.3	1880.63	0.1	0.67	3.1	8.01	0.0	7.99	0.0	-0.01	0.0	2.03	0.1	1.93	0.0	-0.06	0.0
	520	2	37.0	14.5	0	-	335.78	7.7	325.78	1.6	-5.59	3.3	1907.88	6.2	1890.75	9.3	-9.71	2.1	7.94	0.0	7.96	0.0	0.01	0.0	1.79	0.1	1.85	0.1	0.03	0.0
	640	2	37.0	14.4	0	-	336.41	4.2	327.03	6.0	-5.85	1.6	1926.24	17.2	1905.93	0.5	-13.08	12.1	7.90	0.1	7.93	0.0	0.02	0.0	1.64	0.1	1.71	0.0	0.05	0.1
	880	2	37.0	14.4	0	-	324.69	2.7	321.09	1.1	-2.11	0.9	1950.44	8.7	1928.75	5.3	-12.72	8.2	7.83	0.0	7.87	0.0	0.02	0.0	1.44	0.1	1.52	0.1	0.05	0.1
	1120	2	37.0	14.4	0	-	330.78	2.0	324.38	0.0	-4.19	1.6	2005.71	3.1	1975.52	16.6	-19.14	7.1	7.67	0.0	7.73	0.1	0.04	0.0	1.02	0.0	1.16	0.1	0.09	0.1
F. vesiculosus	280	4	35.0	16.1	0.69	0.7	324.69	7.8	377.42	45.7	50.58	10.3	1905.66	14.8	1839.60	42.9	-65.38	16.4	7.96	0.0	8.11	0.1	0.16	0.0	1.90	0.2	2.58	0.4	0.68	0.2
	400	4	35.0	16.4	0.58	0.2	320.39	14.6	378.12	19.2	61.79	11.4	1930.24	9.1	1875.23	17.5	-60.05	26.8	7.89	0.0	8.04	0.0	0.16	0.1	1.69	0.1	2.25	0.1	0.61	0.3
	520	4	35.0	16.1	0.66	0.3	340.16	17.4	389.22	39.2	41.95	5.7	1938.42	4.7	1867.69	26.5	-61.98	2.9	7.87	0.0	8.06	0.1	0.16	0.0	1.62	0.1	2.32	0.2	0.62	0.0
	640	4	35.0	16.0	0.55	0.4	332.11	10.7	390.16	44.9	64.80	17.6	1959.83	2.3	1886.84	42.6	-85.04	24.8	7.82	0.0	8.01	0.1	0.24	0.1	1.43	0.1	2.14	0.4	0.83	0.2
	880	4	35.0	16.0	0.66	0.4	331.95	4.8	375.23	18.8	47.00	13.7	2007.80	4.1	1951.76	23.9	-60.39	18.1	7.67	0.0	7.86	0.1	0.20	0.1	1.06	0.1	1.56	0.2	0.54	0.2
	1120	4	35.0	16.0	0.58	0.3	327.73	3.5	379.61	36.3	52.05	11.3	2025.96	3.8	1960.47	39.9	-68.35	16.2	7.61	0.0	7.83	0.1	0.23	0.0	0.93	0.0	1.49	0.3	0.59	0.1
S. latissima	280	6	36.1	14.8	0.07	0.0	323.91	13.4	341.56	17.6	137.40	71.8	1859.21	57.3	1841.18	77.0	-130.45	158.9	8.05	0.1	8.07	0.2	0.11	0.3	2.25	0.5	2.36	0.8	0.61	1.6
	400	4	36.0	14.3	0.06	0.0	330.62	17.4	344.30	17.3	131.55	87.2	1901.17	19.2	1874.20	31.2	-255.31	80.9	7.97	0.0	8.01	0.1	0.41	0.3	1.86	0.2	2.02	0.2	1.45	1.1
	520	4	36.0	14.3	0.04	0.0	323.44	6.5	333.20	1.5	121.54	46.8	1920.86	14.5	1903.22	16.2	-246.86	87.9	7.92	0.0	7.95	0.0	0.30	0.2	1.68	0.1	1.76	0.1	0.89	0.7
	640	4	36.0	14.2	0.05	0.0	324.06	9.9	336.09	10.6	153.24	70.0	1939.00	15.6	1910.06	21.1	-372.35	153.1	7.87	0.0	7.93	0.0	0.67	0.2	1.53	0.1	1.70	0.1	2.09	0.6
	880	4	36.0	14.3	0.06	0.0	333.05	12.1	348.12	13.7	161.07	62.2	1978.11	4.2	1942.84	17.8	-346.11	130.5	7.76	0.0	7.84	0.0	0.76	0.1	1.22	0.0	1.43	0.1	2.00	0.4
	1120	4	36.0	14.2	0.07	0.0	325.78	12.1	348.98	16.7	224.54	47.3	2016.51	16.0	1969.04	25.8	-441.29	68.2	7.64	0.1	7.77	0.1	1.14	0.1	0.94	0.1	1.22	0.1	2.51	0.1
U. lactuca	280	4	36.0	15.4	0.25	0.1	338.83	24.1	408.44	39.4	155.86	54.7	1866.72	20.4	1770.44	34.2	-229.40	70.1	8.07	0.0	8.25	0.1	0.42	0.1	2.35	0.2	3.22	0.3	2.07	0.6
	400	4	36.0	15.5	0.25	0.1	321.56	9.4	396.33	20.9	164.17	46.3	1912.72	22.9	1837.74	16.3	-162.51	81.8	7.97	0.0	8.12	0.0	0.34	0.2	1.93	0.1	2.59	0.2	1.42	0.6
	520	4	36.0	15.4	0.3	0.1	332.58	7.3	388.83	21.2	104.96	20.4	1929.04	13.2	1851.42	24.8	-146.14	14.8	7.93	0.0	8.10	0.1	0.31	0.0	1.79	0.1	2.46	0.2	1.26	0.2
	640	4	36.0	15.3	0.27	0.1	340.16	8.9	423.98	6.1	183.48	31.3	1951.79	14.7	1844.19	21.2	-232.84	36.1	7.87	0.0	8.11	0.0	0.52	0.1	1.59	0.1	2.52	0.2	2.02	0.3
	880	4	36.0	15.3	0.24	0.1	354.53	19.8	400.08	34.7	113.92	14.8	2001.00	11.9	1931.33	36.6	-161.17	42.0	7.73	0.0	7.91	0.1	0.43	0.1	1.20	0.0	1.74	0.3	1.27	0.3
	1120	4	36.0	15.3	0.29	0.2	328.12	24.7	409.77	37.2	165.96	62.4	2021.60	14.4	1918.30	52.7	-221.27	76.2	7.67	0.0	7.94	0.1	0.60	0.2	1.04	0.1	1.86	0.5	1.73	0.5
Z.marina	280	6	36.9	14.6	0.21	0.1	327.92	6.0	337.60	6.9	28.42	13.2	1910.39	19.9	1889.42	16.7	-61.36	38.0	7.97	0.1	8.02	0.0	0.14	0.1	1.92	0.2	2.11	0.2	0.52	0.3
	400	6	36.9	14.6	0.23	0.1	342.40	14.7	352.97	16.0	27.24	14.4	1923.48	14.4	1902.38	20.6	-50.04	29.0	7.94	0.0	7.99	0.0	0.12	0.1	1.81	0.1	1.99	0.2	0.46	0.2
	520	6	36.9	14.5	0.21	0.1	332.97	3.7	342.97	4.9	25.02	14.2	1935.78	11.7	1919.63	22.7	-45.09	33.1	7.91	0.0	7.95	0.0	0.11	0.0	1.70	0.1	1.84	0.1	0.39	0.1
	640	6	36.9	14.5	0.26	0.1	330.73	5.1	346.56	7.2	36.23	13.7	1951.42	10.9	1923.01	29.2	-63.94	30.8	7.87	0.0	7.94	0.1	0.16	0.0	1.57	0.1	1.81	0.2	0.54	0.1
	880	6	36.9	14.5	0.24	0.1	337.92	12.0	352.55	15.1	35.51	8.0	1991.30	17.2	1958.95	16.0	-80.14	14.1	7.76	0.0	7.85	0.0	0.22	0.1	1.25	0.1	1.51	0.1	0.61	0.2
	1120	6	36.9	14.5	0.33	0.1	332.19	6.6	359.90	13.1	53.72	16.8	2024.79	10.4	1979.76	16.0	-82.67	6.2	7.66	0.0	7.79	0.1	0.25	0.0	1.01	0.1	1.34	0.1	0.60	0.1

Table S2. Summary of the different parameters measured in the residence time and irradiance Experiment 2, done with the sugar kelp *S. latissima*. Values show mean ± SD. DO, dissolved oxygen; DIC, dissolved inorganic carbon; Ω saturation state of calcium mineral (aragonite); PAR, Photosynthetic active radiation.

							DO (μmol L^{-1})			DIC (μmol L^{-1})			pH			Ω_{ar}		
Environmental conditions	Water flow	Treatment	PAR (μmol $m^{-2} s^{-1}$)	Sugar kelp biomass (g DW)	Sal.	Temp.	Inflow	Outflow	ΔDO	Inflow	Outflow	ΔDIC	Inflow	Outflow	ΔpH	Inflow	Outflow	ΔΩ
Ambient	High (1.4 L min^{-1})	Control	6.33	0 -	30.0	11.3	337.83 -	NA -	NA -	2111.27 -	NA -	NA -	7.84 -	NA -	NA -	1.23 -	NA -	NA -
			19.38	0 -	30.0	11.2	336.27 -	340.33 -	4.06 -	2104.87 -	2106.30 -	1.43 -	7.86 -	7.86 -	0.00 -	1.29 -	1.27 -	-0.01 -
			75.48	0 -	29.9	11.2	337.83 -	341.27 -	3.44 -	2106.38 -	2105.38 -	-1.00 -	7.86 -	7.86 -	0.00 -	1.27 -	1.28 -	0.01 -
			144.37	0 -	30.0	11.2	339.71 -	344.08 -	4.38 -	2099.85 -	2101.36 -	1.50 -	7.88 -	7.87 -	0.00 -	1.33 -	1.31 -	-0.01 -
			207.93	0 -	30.1	11.2	340.02 -	345.33 -	5.31 -	2100.82 -	2099.31 -	-1.51 -	7.87 -	7.88 -	0.01 -	1.32 -	1.33 -	0.01 -
			258.48	0 -	30.1	11.2	340.33 -	346.90 -	6.56 -	2099.74 -	2099.97 -	0.23 -	7.88 -	7.88 -	0.00 -	1.33 -	1.32 -	0.00 -
		Kelp	6.33	18.47 0.2	30.0	11.4	337.83 -	333.98 0.9	-3.85 0.9	2111.27 -	2107.52 1.1	-3.74 0.0	7.84 -	7.85 0.0	0.01 0.0	1.23 -	1.26 0.0	0.03 0.0
			19.38	18.47 0.2	30.0	11.2	336.27 -	338.98 0.7	2.71 0.7	2104.87 -	2102.47 2.0	-2.40 0.0	7.86 -	7.87 0.0	0.01 0.0	1.29 -	1.30 0.0	0.02 0.0
			75.48	18.47 0.2	29.9	11.2	337.83 -	345.08 1.5	7.25 1.5	2106.38 -	2100.84 0.6	-5.54 0.0	7.86 -	7.88 0.0	0.02 0.0	1.27 -	1.32 0.0	0.05 0.0
			144.37	18.47 0.2	30.0	11.2	339.71 -	352.27 2.0	12.56 2.0	2099.85 -	2093.55 1.3	-6.31 0.0	7.88 -	7.90 0.0	0.02 0.0	1.33 -	1.38 0.0	0.05 0.0
			207.93	18.47 0.2	30.0	11.3	340.02 -	355.02 1.9	15.00 1.9	2100.82 -	2093.57 0.5	-7.25 0.0	7.87 -	7.90 0.0	0.03 0.0	1.32 -	1.38 0.0	0.06 0.0
			258.48	18.47 0.2	30.1	11.3	340.33 -	356.58 2.2	16.25 2.2	2099.74 -	2092.17 0.7	-7.57 0.0	7.88 -	7.90 0.0	0.02 0.0	1.33 -	1.39 0.0	0.07 0.0
	Low (0.5 L min^{-1})	Control	6.33	0 -	30.0	11.4	337.83 -	NA -	NA -	2111.27 -	NA -	NA -	7.84 -	NA -	NA -	1.23 -	NA -	NA -
			19.38	0 -	30.0	11.4	336.27 -	340.33 -	4.06 -	2104.87 -	2103.73 -	-1.14 -	7.86 -	7.86 -	0.00 -	1.29 -	1.29 -	0.01 -
			75.48	0 -	29.9	11.3	337.83 -	NA -	NA -	2106.38 -	NA -	NA -	7.86 -	NA -	NA -	1.27 -	NA -	NA -
			144.37	0 -	29.9	11.5	339.71 -	346.58 -	6.88 -	2099.85 -	2102.25 -	2.40 -	7.88 -	7.87 -	-0.01 -	1.33 -	1.31 -	-0.02 -
			207.93	0 -	30.0	11.5	340.02 -	347.52 -	7.50 -	2100.82 -	2097.19 -	-3.62 -	7.87 -	7.88 -	0.01 -	1.32 -	1.35 -	0.03 -
			258.48	0 -	30.1	11.4	340.33 -	353.46 -	13.13 -	2099.74 -	2095.32 -	-4.42 -	7.88 -	7.89 -	0.01 -	1.33 -	1.37 -	0.04 -
		Kelp	6.33	18.16 0.4	30.0	11.5	337.83 -	332.44 0.9	-5.39 0.9	2111.27 -	2110.50 5.6	-0.77 0.0	7.84 -	7.84 0.0	0.00 0.0	1.23 -	1.24 0.0	0.01 0.0
			19.38	18.16 0.4	29.9	11.4	336.27 -	338.15 0.9	1.88 0.9	2104.87 -	2102.72 2.5	-2.15 0.0	7.86 -	7.87 0.0	0.01 0.0	1.29 -	1.30 0.0	0.02 0.0
			75.48	18.16 0.4	29.8	11.4	337.83 -	348.22 2.3	10.39 2.3	2106.38 -	2094.99 0.4	-11.39 0.0	7.86 -	7.89 0.0	0.04 0.0	1.27 -	1.37 0.0	0.10 0.0
			144.37	18.16 0.4	29.9	11.3	339.71 -	358.46 4.1	18.75 4.1	2099.85 -	2086.30 1.8	-13.56 0.0	7.88 -	7.92 0.0	0.04 0.0	1.33 -	1.44 0.0	0.12 0.0
			207.93	18.16 0.4	30.0	11.4	340.02 -	365.84 4.4	25.81 4.4	2100.82 -	2081.38 2.4	-19.43 0.0	7.87 -	7.93 0.0	0.06 0.0	1.32 -	1.49 0.0	0.17 0.0
			258.48	18.16 0.4	30.1	11.4	340.33 -	366.02 6.7	25.69 6.7	2099.74 -	2080.24 3.9	-19.50 0.0	7.88 -	7.93 0.0	0.05 0.0	1.33 -	1.50 0.0	0.17 0.0

							DO (μmol L^{-1})			DIC (μmol L^{-1})			pH			Ω_{ar}		
Environmental conditions	Water flow	Treatment	PAR (μmol $m^{-2} s^{-1}$)	Sugar kelp biomass (g DW)	Sal.	Temp.	Inflow	Outflow	ΔDO	Inflow	Outflow	ΔDIC	Inflow	Outflow	ΔpH	Inflow	Outflow	ΔΩ
Future	High (1.4 L min^{-1})	Control	6.33	0.00 -	30.1	12.4	325.96 -	336.58 -	10.63 -	2238.66 -	2243.77 -	5.11 -	7.23 -	7.37 -	-0.01 -	0.48 -	0.46 -	-0.02 -
			19.38	0.00 -	30.2	12.4	340.02 -	NA -	NA -	2241.59 -	NA -	NA -	7.13 -	NA -	NA -	0.47 -	NA -	NA -
			75.48	0.00 -	30.0	12.4	333.15 -	337.83 -	4.69 -	2240.8 -	2229.59 -	-11.21 -	7.29 -	7.42 -	0.04 -	0.47 -	0.51 -	0.04 -
			144.37	0.00 -	30.1	12.3	336.58 -	339.71 -	3.13 -	2240.93 -	2220.08 -	-20.85 -	7.55 -	7.45 -	0.07 -	0.47 -	0.55 -	0.08 -
			207.93	0.00 -	30.2	12.4	335.96 -	342.21 -	6.25 -	2240.56 -	2266.04 -	25.48 -	7.55 -	7.30 -	-0.08 -	0.47 -	0.39 -	-0.08 -
			258.48	0.00 -	30.2	12.5	337.21 -	340.96 -	3.75 -	2240.32 -	2246.85 -	6.53 -	7.51 -	7.36 -	-0.02 -	0.47 -	0.45 -	-0.02 -
		Kelp	6.33	19.30 0.7	30.1	12.5	325.96 -	334.50 3.3	8.54 3.3	2238.66 -	2261.46 9.4	22.8 0.0	7.23 -	7.31 0.0	-0.07 0.0	0.48 -	0.41 0.0	-0.08 0.0
			19.38	19.30 0.7	30.1	12.4	340.02 -	338.61 0.5	-1.41 0.5	2241.59 -	2282.33 47.4	40.74 0.0	7.13 -	7.26 0.1	-0.12 0.1	0.47 -	0.37 0.1	-0.1 0.1
			75.48	19.30 0.7	30.0	12.4	333.15 -	344.52 1.5	11.38 1.5	2240.8 -	2227.38 5.6	-13.42 0.0	7.29 -	7.42 0.0	0.05 0.0	0.47 -	0.52 0.0	0.05 0.0
			144.37	19.30 0.7	30.1	12.4	336.58 -	351.96 2.9	15.38 2.9	2240.93 -	2214.35 8.1	-26.58 0.0	7.55 -	7.47 0.0	0.09 0.0	0.47 -	0.58 0.0	0.1 0.0
			207.93	19.30 0.7	30.2	12.4	335.96 -	356.27 3.8	20.31 3.8	2240.56 -	2220.04 17.6	-20.51 0.0	7.55 -	7.45 0.1	0.07 0.1	0.47 -	0.56 0.1	0.08 0.1
			258.48	19.30 0.7	30.2	12.5	337.21 -	356.83 4.7	19.63 4.7	2240.32 -	2228.47 31.6	-11.85 0.0	7.51 -	7.42 0.1	0.04 0.1	0.47 -	0.53 0.1	0.06 0.1
	Low (0.5 L min^{-1})	Control	6.33	0.00 -	30.2	12.4	325.96 -	334.71 -	8.75 -	2238.66 -	2264.49 -	25.82 -	7.23 -	7.30 -	-0.08 -	0.48 -	0.40 -	-0.08 -
			19.38	0.00 -	30.2	12.4	340.02 -	NA -	NA -	2241.59 -	NA -	NA -	7.13 -	NA -	NA -	0.47 -	NA -	NA -
			75.48	0.00 -	30.0	12.4	333.15 -	NA -	NA -	2240.8 -	NA -	NA -	7.29 -	NA -	NA -	0.47 -	NA -	NA -
			144.37	0.00 -	30.1	12.4	336.58 -	343.15 -	6.56 -	2240.93 -	2242.72 -	1.79 -	7.55 -	7.37 -	-0.01 -	0.47 -	0.47 -	-0.01 -
			207.93	0.00 -	30.2	12.4	335.96 -	346.27 -	10.31 -	2240.56 -	2237.92 -	-2.64 -	7.55 -	7.39 -	0.01 -	0.47 -	0.48 -	0.01 -
			258.48	0.00 -	30.3	12.4	337.21 -	343.77 -	6.56 -	2240.32 -	2244.23 -	3.91 -	7.51 -	7.36 -	-0.01 -	0.47 -	0.46 -	-0.01 -
		Kelp	6.33	17.95 0.8	30.2	12.5	325.96 -	331.48 2.9	5.52 2.9	2238.66 -	2249.56 14.0	10.9 0.0	7.23 -	7.35 0.1	-0.03 0.1	0.48 -	0.45 0.1	-0.04 0.1
			19.38	17.95 0.8	30.1	12.5	340.02 -	340.65 1.0	0.63 1.0	2241.59 -	2266.44 26.6	24.86 0.0	7.13 -	7.30 0.1	-0.08 0.1	0.47 -	0.40 0.1	-0.07 0.1
			75.48	17.95 0.8	30.0	12.5	333.15 -	352.08 3.4	18.94 3.4	2240.8 -	2227.72 19.9	-13.08 0.0	7.29 -	7.42 0.1	0.05 0.1	0.47 -	0.53 0.1	0.05 0.1
			144.37	17.95 0.8	30.1	12.4	336.58 -	363.46 4.7	26.88 4.7	2240.93 -	2219.56 11.0	-21.37 0.0	7.55 -	7.45 0.0	0.07 0.0	0.47 -	0.56 0.1	0.08 0.1
			207.93	17.95 0.8	30.2	12.5	335.96 -	369.77 4.0	33.81 4.0	2240.56 -	2206.45 8.1	-34.11 0.0	7.55 -	7.49 0.0	0.12 0.0	0.47 -	0.61 0.0	0.14 0.0
			258.48	17.95 0.8	30.2	12.6	337.21 -	375.46 8.6	38.25 8.6	2240.32 -	2222.76 11.8	-17.55 0.0	7.51 -	7.44 0.0	0.06 0.0	0.47 -	0.54 0.1	0.07 0.1

Table S3. Coefficients of the linear models used in the statistical data analysis in Experiment 1 for the comparison among marine macrophyte species. Summary output from linear models as reported in the R software. Estimates and SE are provided, where SE is within (). From these estimates, the intercepts and slopes of each linear interaction can be calculated. Intercepts of each species should be calculated by adding them to the intercept of the control (row Intercept). Slopes of each species should be calculated by adding them to the slope of the control (row pCO2). n.s, non-significant; N, number of samples; R2, r-squared of linear model. DO, dissolved oxygen; DIC, dissolved inorganic carbon; Ω saturation state of calcium mineral (aragonite).

Comparison among species

	DO	DIC	pH	Ω
Intercept	-21.02	9.29	-0.04	-0.18
	(13.56)	(61.81)	(0.12)	(0.72)
F. vesiculosus	50.64	-77.72	0.16	1.40
	(13.25)	(77.69)	(0.15)	(0.90)
S. latissima	155.37	-115.11	-0.18	0.71
	(12.79)	(72.83)	(0.14)	(0.85)
U.lactuca	149.26	-184.77	0.27	2.94
	(13.00)	(75.45)	(0.14)	(0.88)
Z.marina	35.04	-35.90	0.046	0.67
	(12.20)	(73.60)	(0.14)	(0.85)
pCO2	0.03	-0.03	0.00	0.00
	(0.01)	(0.10)	(0.00)	(0.00)
F. vesiculosus :pCO2	n.s	0.04	0.00	-0.00
		(0.12)	(0.00)	(0.00)
S.latissima :pCO2	n.s	-0.27	0.01	0.00
		(0.12)	(0.00)	(0.00)
U.lactuca :pCO2	n.s	0.01	0.00	-0.00
		(0.12)	(0.00)	(0.00)
Z.marina :pCO2	n.s	-0.02	0.00	0.00
		(0.12)	(0.00)	(0.00)
N	124	124	124	124
R2	0.70	0.67	0.72	0.55
