# Supplementary material for: Optimizing marine macrophyte capacity to locally ameliorate ocean acidification under variable light and flow regimes: Insights from an experimental approach
Source: PLoS One. 2023 Oct 11;18(10):e0288548. doi: 10.1371/journal.pone.0288548 (PMC10566731; doi:10.1371/journal.pone.0288548)
Supplement: Attachment — Submitted filename: Comments-to-the-author.pdf [file pone.0288548.s006.pdf]

Comments to the author:

The manuscript *Optimizing marine macrophyte capacity to locally ameliorate ocean acidification under variable light and flow regimes: Insights from an experimental approach* assessed biotic (i.e., species identity) and abiotic drivers (i.e., light level, residence time) of macrophytes' ability to ameliorate seawater acidity under ambient and increased pCO_2 conditions. The study had three main goals: 1) to compare the ability of four macrophyte species to ameliorate seawater acidity under a range of pCO_2 conditions (Experiment 1), 2) to assess the effects of light level and residence time on the best-performing species' ability to ameliorate seawater acidity under ambient and future pCO_2 and temperature conditions (Experiment 2), and 3) to create a model predicting amelioration effects as a function of light level and flow rate.

In Experiment 1, the authors identified *Saccharina latissima* as the best-performing species in its ability to ameliorate seawater acidity (i.e., consume dissolved inorganic carbon (DIC), increase pH, and increase aragonite saturation state) under increased pCO_2 conditions. In Experiment 2, the authors found that *S. latissima* amelioration effects (relative to control treatments without *S. latissima*) were greater under future compared to ambient pCO_2 and temperature conditions. They also found stronger amelioration effects when residence time was high (or flow rate was low) under ambient conditions, but residence time had no effect under future conditions. The authors interpret this as evidence that as macrophytes become pCO_2-saturated, residence time may no longer be a relevant driver of macrophyte seawater acidity amelioration. The predictive model was parameterized by Experiment 2 and predicted amelioration effects via DIC uptake by *S. latissima* as a function of light level and flow rate.

This work advances discussion on the role of macrophytes in mitigating ocean acidification by evaluating species differences and effects of light and residence time on seawater acidity amelioration in tightly controlled laboratory experiments. Overall, the manuscript is well written, and the study objectives are clear. However, there are some aspects of the experimental design and methodology that should be clarified, and the limitations to interpretation and model extrapolation of results obtained from the laboratory-based studies warrant further discussion.

Major comments:

1. In Experiment 1, I am not sure if an averaged value for total alkalinity for each species and pCO_2 level treatment combination is appropriate to use in estimating other carbonate chemistry parameters used as response variables (line 223). While total alkalinity did not vary much between replicates, using an average value for a subset of replicates within the same treatment combination 'hides' within-group variability and may be a form of pseudoreplication. If this cannot be properly justified, it may be more appropriate to run the statistical analyses with the three replicates per treatment combination where total alkalinity was actually measured.
2. In Experiment 2, the future conditions included increased temperature by 2°C but there is little attention given to the effects of warming throughout the manuscript. It would be helpful to provide context on whether this is a modest increase in temperature in terms of *S. latissima* physiology and whether 13°C falls within its tolerance range.
3. There is also no mention of whether the elevated temperature in the future conditions in Experiment 2 could have confounded the effects of residence time and pCO_2 levels. The treatments were not independent (i.e., there were no treatments with ambient pCO_2 +

future temperature or treatments with future pCO_2 + ambient temperature), so while the ambient and future conditions may reflect the most realistic combinations of pCO_2 levels and temperatures, it's not possible to rule out a temperature effect here.

4. The predictive model is a bit unclear, so it may be helpful to provide a written formulation of the model. In some parts of the manuscript, the model seems to include biomass as a predictor variable (line 39) but in others, the response variable appears to be standardized by biomass (lines 294-295). If the response variable is change in DIC per gram of biomass, then biomass is not really a predictor in the model but rather the unit of measurement for the response variable. Scaling the response variable by biomass relies on the assumption that metabolism (e.g., DIC uptake) scales linearly with biomass.
5. There could be more discussion on the ecosystem-level implications of the laboratory-based findings. In natural settings, seawater acidity amelioration by macrophytes likely varies over space and time. The authors touched on how basin geomorphology, diurnal cycles, and water clarity will affect the ability of *S. latissima* to ameliorate seawater acidity. If *S. latissima* produces the greatest amelioration effects under saturating light conditions compared to the other species, are these effects countered by stronger respiration effects, potentially increasing seawater acidity, under dark conditions? If so, I think it is important to caveat that *S. latissima* may not be the ideal candidate for acidification mitigation. Additionally, other processes occurring in the water column or benthos (e.g., alkalinity production/consumption related to respiration, air-water CO_2 exchange, etc.) can affect acidity. How might these uncertainties affect the reliability of the predictive model?

line 106: it may be helpful to define residence time.

lines 130-132: alternation of generations is not particularly useful to include here since it is common in most plants and algae to some degree.

lines 149-150: how did you ensure equilibrium was reached before starting the experiment?

lines 153-154: what was the flow rate?

lines 164-165: how did temperature variation throughout the incubation affect carbonate chemistry calculations?

lines 166: maybe state upfront the measured carbonate chemistry parameters.

line 179: maybe explain that the pH controller system could maintain a pCO_2 level within a range of 200 µatm.

lines 199-200: how did you ensure equilibrium was reached before starting the experiment?

lines 284-286: it's not clear how the same light curves approach was applied to the other response variables. In equations (1) and (2), were P and Pmax terms simply replaced by net ΔDIC/ΔpH/$\Delta\Omega$ between tank inflow and outflow and the maximum ΔDIC/ΔpH/$\Delta\Omega$ at saturating irradiance, respectively? I suggest writing out the equations in a more generalized form for

clarity. For example, P and Pmax could be replaced with other terms, such as R and Rmax, and state that R is $\Delta DO/\Delta DIC/\Delta pH/\Delta\Omega$.

line 292: was leaf surface area accounted for as well?

line 305: it's best practice to state R version used for analyses.

lines 328-330: should the values mentioned here match the slopes presented in Table S3? If not, where do these numbers come from?

lines 334-339: should the values mentioned here match the slopes presented in Table S3? If not, where do these numbers come from?

lines 357-359: was the higher impact of kelp in future compared ambient conditions assessed qualitatively?

lines 446-447: since only two flow rates (0.02 and 0.06 cm s^{-1}) were used to parameterize the model, are there limitations when interpreting extrapolated flows an order of magnitude higher?

line 469: in the example starting on line 469, the predictive model is applied to an example. The spatial extent of the effect is noted (1000 m^3), but what about the temporal extent, i.e., how long would the effect be sustained?

lines 496-503: mention that findings are limited to macrophyte seawater acidity amelioration during photosynthesis in a laboratory setting, in the absence of air-water exchange, etc.

Table 1: label experiments numerically, e.g., 'Experiment 1 – Comparison among species' and include statistical analyses used, either in the table or table caption. If ANOVA was used to compare overall effects of species, pCO_2, and their interaction for Experiment 1, this should be listed in the methods, too.

Table S1: since the number of replicates per treatment combination (n) varied, consider including n for each treatment combination in this table.

Table S3: how was the linear model constructed so that coefficients were available for each species? I understand that an intercept-free model in lm() can accomplish this, but an intercept is reported in this table. More description in the methods would make the data analyses more reproducible.
